# Synthesis and Characterization of Microcapsules as Fillers for Self-Healing Dental Composites

**DOI:** 10.3390/nano14221853

**Published:** 2024-11-20

**Authors:** Maria Amalia Tăut, Marioara Moldovan, Miuţa Filip, Ioan Petean, Codruţa Saroşi, Stanca Cuc, Adrian Catalin Taut, Ioan Ardelean, Viorica Lazăr, Sorin Claudiu Man

**Affiliations:** 1 Physics and Chemistry Department, Technical University of Cluj-Napoca, 103-105 Bulevardul Muncii Street, 400641 Cluj-Napoca, Romania; amalia.taut@chem.utcluj.ro (M.A.T.); ioan.ardelean@phys.utcluj.ro (I.A.); 2 Raluca Ripan Institute for Research in Chemistry, Babeș-Bolyai University, 30 Fantanele Street, 400294 Cluj-Napoca, Romania; marioara.moldovan@ubbcluj.ro (M.M.); miuta.filip@ubbcluj.ro (M.F.); codruta.sarosi@ubbcluj.ro (C.S.); 3 Faculty of Chemistry and Chemical Engineering, Babes-Bolyai University, 11 Arany Janos Street, 400028 Cluj-Napoca, Romania; petean.ioan@gmail.com; 4 Applied Electronics Department, Technical University of Cluj-Napoca, 26-28 George Barițiu Street, 400027 Cluj-Napoca, Romania; adrian.taut@ael.utcluj.ro; 5 Department of General Medicine, Vasile Goldis University of Medicine, 310048 Arad, Romania; docleordean@yahoo.com; 6 Pediatric Clinic II, Clinical Hospital Emergency of Arad County, 310037 Arad, Romania; 7 Department of Pediatrics, “Iuliu Hațieganu” University of Medicine and Pharmacy Cluj-Napoca, 400124 Cluj-Napoca, Romania; sorinman@gmail.com

**Keywords:** self-healing, microcapsule, microstructure, progressive release

## Abstract

This article proposes the synthesis and characterization of (triethylene glycol dimethacrylate–N,N-dihydroxyethyl-p-toluidine) TEGDMA-DHEPT self-healing microcapsules for their inclusion in dental composite formulations. The obtaining method is the in situ emulsion polymerization of the (poly urea-formaldehyde) (PUF) coatings. The microcapsules were characterized by Fourier transform infrared spectroscopy (FTIR), scanning electron microscopy (SEM), atomic force microscopy (AFM), high-performance liquid chromatography (HPLC), and low-field nuclear magnetic resonance (NMR) techniques. The optimal formation of uniform microcapsules is achieved at a stirring speed of 800 rpm and centrifugation is no longer necessary. HPLC demonstrates that the microcapsules formed at 800 rpm show a better control of liquid release than the heterogeneous ones obtained at a lower stirring speed. The centrifuged samples have rounded shapes, with dimensions between 80 and 800 nm, while the non-centrifuged samples are more uniform, with a spherical shape and dimensions of approximately 800 nm.

## 1. Introduction

Resin composites are commonly used for filling dental cavities due to their excellent aesthetics and ability to be applied directly [1,2]. The development of dental composites requires the careful selection and combination of different materials to obtain key properties, such as mechanical strength, biocompatibility, and long-term durability [3]. The development of hybrid-modified composites improves the performance of individual components, as it is not feasible to create a single material that incorporates all of the desired characteristics [4]. However, the longevity of these restorations is limited, with half failing within ten years. The primary reason for the failure of composite restorations is the development and accumulation of micro-cracks, leading to fractures. These cracks result from masticatory forces and thermal stress [5], which also facilitate the development of secondary caries. Recent research indicates that fractures are a common cause of composite failure, particularly within the first five years, while secondary caries become more prevalent after five years [6].

One of the major limitations of these composites is their susceptibility to micro-cracks, which can complicate the restoration process and significantly reduce their reliability and lifespan. Self-healing techniques have emerged as a promising solution to mitigate these issues and extend the material’s service life without compromising its mechanical properties.

Unfortunately, there is limited research on the advantages and challenges associated with the self-healing properties of different matrix materials. This gap presents an opportunity for innovation and exploration. Essentially, self-healing materials can recover their original characteristics after damage, particularly their mechanical properties. The microcapsule-based self-healing mechanisms are highly promising, with composites repaired in this way potentially performing better than those repaired by traditional methods.

In recent decades, researchers have explored various systems, such as incorporating monomer-filled tubes, glass fibers, and capsules into host materials to release the monomer upon crack initiation, thereby autonomously healing the material. One of the most advanced self-healing systems was developed by White et al. (2001), which involves microcapsules with a urea-formaldehyde (UF) coating that incorporates dicyclopentadiene (DCPD) as a regenerating agent. These microcapsules are embedded in an epoxy matrix with a selective catalyst. When a crack occurs, the microcapsule shell ruptures, releasing DCPD, which reacts with the catalyst to bridge the crack [7].

The most used materials for obtaining the microcapsule shells are poly(urea-formaldehyde) (PUF) [8], poly (melamine-formaldehyde (PMF) [9], polymethyl methacrylate (PMMA) [10], and polyurethane (PU) [11]. The core materials, including the healing agents, can be dicyclopentadiene (DCPD) [12], classical alicyclic derivatives [13], dry oils [14], polymerizable agents [15], organic solvents [16], and epoxy resins [17].

There are three main methods for obtaining microcapsules, depending on the method used to create their shell. Physical methods can be spray drying [18] or solvent evaporation [19]. Chemical methods include in situ polymerization [20], interfacial polymerization [21], or piercing [22]. Physicochemical methods [23] include oil phase separation, simple coacervation, or complex coacervation methods. The most efficient method for the preparation of self-healing microcapsules is considered to be in situ polymerization. It is an easy method that does not require complicated equipment. The size of the microcapsules can be easily controlled by adjusting the stirring speed. The method has two stages: obtaining an oil-in-water emulsion and forming the shell for encapsulation. In the first stage, the healing oil is emulsified by mechanical or ultrasonic agitation using an emulsifier, and the healing oil is dispersed in small drops. The stirring speed, emulsifier amount, and emulsifier type influence the shape and size of the microcapsules. The second stage forms the shell that encapsulates the healing liquid. The polymerization of the monomers or their pre-polymerization takes place with the catalysts on the surface of the droplets. The choice of shell material, namely the core/shell ratio or the stirring speed, can influence the encapsulation efficiency of the microcapsules [24].

Most of the self-healing composites are based on the microcapsule system, which are embedded beside nano-filler particles. Each of the microcapsules has an outer shell that encapsulates a regenerating liquid. The shell rupture is initiated when the microcapsule collides with damages and cracks on the tooth surface, starting the progressive release of the regenerating fluid, which penetrates into the crack planes and the damaged site, where it reacts with the embedded catalyst. Self-healing occurs as the regenerating agent polymerizes, fills, and binds the cracks and prevents further propagation [25].

This study proposes the synthesis and characterization of self-healing microcapsules for use in dental composite formulations. We opted for the synthesis of (triethylene glycol dimethacrylate–N,N-dihydroxyethyl-p-toluidine) TEGDMA-DHEPT microcapsules, which include the optimization of the in situ emulsion polymerization of (poly urea-formaldehyde) PUF shells. The shape and size of the microcapsules depend on the stirring speed, and the centrifugation and filtration processes. Therefore, we aim to vary these parameters to obtain the best configuration for the obtained microcapsules. The complex physicochemical characterization of the obtained samples relies on FTIR spectroscopy to unveil the chemical bonds within the samples. The microstructural aspects are revealed by scanning electron microscopy (SEM) and the ultrastructural details are revealed by atomic force microscopy (AFM). The incorporation of the healing polymer is investigated via nuclear magnetic resonance (NMR) relaxometry [26]. The optimal synthesis parameters will be established based on the physicochemical results. The progressive release of the active compound is followed by HPLC spectroscopy. The incorporation of the synthesized microcapsules into dental resin composites will be discussed in our future work.

## 2. Materials and Methods

### 2.1. Microcapsules Preparation

The microcapsule shells were composed of urea (CH_4_N_2_O 99%), ammonium chloride (NH_4_Cl 99%), resorcinol (C_6_H_6_O_2_ 99%), and a 37% aqueous formaldehyde solution. The healing agent was a mixture of N,N-dihydroxyethyl-p-toluidine (DHEPT 90%) and triethylene glycol dimethacrylate (TEGDMA 95%). Polyvinyl alcohol (PVA degree of hydrolysis 87–89%) was used as a surfactant, while hydrochloric acid (HCl) and 1M sodium hydroxide (NaOH 98%) were used as pH regulators. All chemicals were purchased from Sigma Aldrich (Chemie GmbH, Steinheim, Germany).

The microcapsules were prepared via in situ polymerization in an oil-in-water (O/W) emulsion. The polymerization components were added to a Berzelius glass at room temperature: 50 mL of distilled water and 13 mL of 2.5% PVA aqueous solution, prepared the day before.

**Figure 1:**
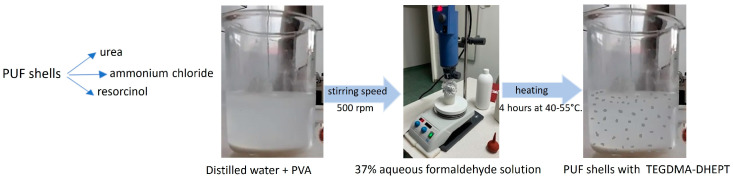
Obtaining microcapsules via in situ polymerization in an oil-in-water (O/W) emulsion.

Figure 1 shows the process of obtaining self-healing capsules.

A magnetic stirrer mixed the solution. The coating materials—1.25 g urea, 0.15 g ammonium chloride, and 0.15 g resorcinol—were then added. Once dissolved, the pH was adjusted to 3.5 with HCl or NaOH. The stirring speed was increased to 500 rpm and 30 mL of healing liquid, prepared the previous day, was added drop-wise. This healing liquid comprised TEGDMA monomer and 1% of DHEPT amine by weight. The mixture was stirred for 10 min until an emulsion formed. Subsequently, 3.2 g of 37% formaldehyde aqueous solution was added and the glass was covered with aluminum foil to prevent evaporation. The synthesis parameters were varied according to the data in Table 1.

### 2.2. Investigation Methods

The chemical bonds within samples were investigated by Fourier Transformed Infrared spectroscopy (FTIR) using an FTIR 610 spectrometer (Jasco Corporation, Tokyo, Japan), equipped with an attenuated total reflectance (ATR) attachment with a horizontal ZnSe crystal (Jasco PRO400S) in the 4000–500 cm^−1^ wave number range. The resolution of the spectra was 4 cm^−1^, and scans were repeated of 100 times. The FTIR spectra were registered at room temperature.

Scanning Electron Microscopy (SEM) was done with an Inspect™ SEM microscope (FEI Company, Hillsboro, OR, USA), which was operated in the low-vacuum mode using an acceleration voltage of 20 kV.

The Atomic Force Microscopy (AFM) was performed with a JEOL scanning probe microscope, JSPM 4210, produced by JEOL Company, Japan, Tokyo. The surface of the nanocapsule samples was tapped using NSC 15 cantilevers produced by MikroMasch, Estonia, Tallinn (resonant frequency of 325 kHz; force constant of 40 N/m). The topographic images were scanned at different areas for at least three different macroscopic zones in order to ensure a proper statistical observation. The scan rate is adapted to the scanned area and local topography; thus, it ranges from about 1.5 to 3 Hz, depending on the scanned area and local samples characteristics. The obtained images were analyzed with the specialized software Jeol WIN SPM 2.0, produced by JEOL, Japan, Tokyo. The topographic features and surface roughness parameters were followed. The mean roughness values were determined for each sample by at least three independent measurements.

High-performance liquid chromatography (HPLC) was employed for the controlled liquid release assessment after 7 and 14 days. The analyses were performed on an HPLC Chromatograph (Jasco Tokyo, Japan). The filtered microcapsules were stored in glass vials for 7 and 14 days. 100 mg of these microcapsules were taken after each time interval and dispersed into bi-distilled water at a concentration of 2 mg/mL. The dispersions were agitated for 5 min for the healing agent release into the water, followed by 5 min of resting. The supernatant was filtered and injected into the HPLC.

Low-field nuclear magnetic resonance (NMR) techniques were used to reveal the presence of the liquid component inside the sample and to estimate the liquid-to-solid ratio in a similar manner to Ref. [26]. Thus, the presence of the liquid component in the sample was identified on the basis of relaxation time distributions determined via the Carr–Purcell–Meiboom–Gill (CPMG) technique [27], combined with a numerical inverse Laplace transformation [28]. The liquid-to-solid ratios were estimated using the free induction decay (FID) signal recorded immediately following a hard 90-degree radiofrequency pulse [24]. The investigations were performed at 35 °C, using a low-field NMR instrument (MINISPEC MQ 20, Bruker, Karlsruhe, Germany) operating at 20 MHz proton resonance frequency.

## 3. Results

### 3.1. Fourier-Transform Infrared Spectroscopy (FTIR)

**Figure 2:**
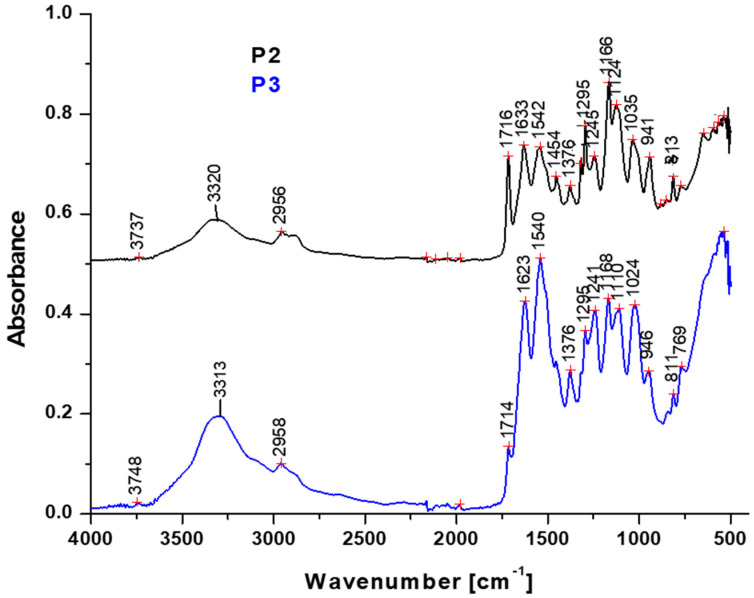
FTIR spectra of P2 and P3 samples.

The FTIR spectra results for the prepared microcapsules are presented in Figure 2. Their chemical bonds interact with the infrared beam inducing specific vibrations such as stretching and bending that generate specific absorption bands.

Both samples P2 and P3 have shown the same absorption bands, proving their similar chemical structures. However, it was noticed that the absorption bands for C=C stretching within DHEPT and O-H bending due to water absorption are more intense in P3 than in P2, along with O-H stretching. This indicates strong evidence for the active compound within the microcapsule core in P3.

The FTIR spectra of the P2 and P3 samples show peaks of 3320/3316 cm^−1^ that correspond to O-H stretching [29]. Two bands at 2956/2958 cm^−1^ and 2825 cm^−1^ were observed, corresponding to the asymmetric and symmetric stretching vibrations of -CH2_2_-CH_3_ groups [30,31,32]. The specific bands of 1714/1716 cm^−1^ and 1540/1542 cm^−1^ are assigned to the ester C=O and aromatic C=C stretching vibrations from the TEGDMA compound [33,34]. In the region of 1500–700 cm^−1^, one can observe the vibrations due to the stretching of groups of methoxy O-CH_3_, ether C-O-C, and the C=C stretching of the aromatic ring, in either the DHEPT compound or in the TEGDMA-containing compounds [34,35]. The characteristic peaks at 1454 cm^−1^ and 1035/1024 cm^−1^ can be assigned to C-N stretching and NH_2_ bending vibrations from the urea compound [36,37]. The vibrations of 811/813 cm^−1^ correspond to the out-of-plane C-H bending vibration of the studied compounds [32].

### 3.2. Assessment of Micro and Nanostructures

**Figure 3:**
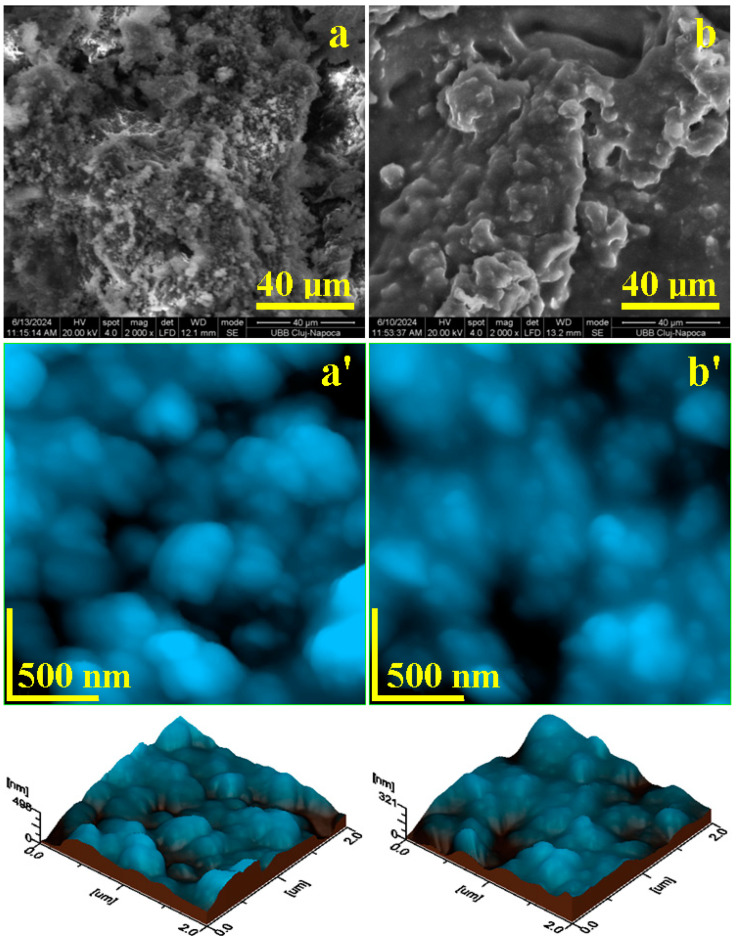
Microstructural investigation of nanocapsules after synthesis: SEM images of samples P1 (**a**) and P2 (**b**). Microstructure accompanied by ultrastructure as revealed by AFM: samples P1 (**a’**) and P2 (**b’**). Topographic 3D profile is presented below each AFM image.

P1 nanocapsules appear as small spots having a coalescence tendency, as observed in the SEM image shown in Figure 3a. These are well-individualized and appear as small spots deposited over the microstructural features with sizes of about 40 µm and relatively irregular shapes. The consistency of the microstructural domains is caused by the physical attraction between the adjacent nanocapsules rather than by the chemical bonds, which is a fact sustained by the FTIR spectra.

The diameter of the nanocapsules is difficult to assess from the SEM image, therefore the AFM investigation was performed on a scanned area of 2 µm × 2 µm, as seen in Figure 3a’. The obtained topographic image reveals well-individualized nanocapsules with rounded shapes and diameters ranging from 80 to 350 nm. These nanocapsules are clustered close to each other without bonding fusion, which allow us to consider that the observed ultrastructure is a dense population of individualized parts and not a small micro-sized cluster. It is further expected that an intense physical treatment will bring more liberty for each of the observed nanocapsules. The topography of the nanocapsule ultrastructural organization is observed better in the 3D profile presented beneath the topographic image. The well-individualized nanocapsules can be clearly observed and their presence induces a mean roughness of Ra = 59.7 nm and Rq = 76.7 nm.

The P2 sample presents a dendritic microstructure formed by interlaced domains with a length of about 80–90 µm and a width around 40–50 µm, as seen in Figure 3b. These microstructural features contain well-individualized nanocapsules, which are held together by a thin layer of dispersion environment that remains attached on their surface, which is a fact confirmed by the FTIR spectra. The nanocapsules appear as small, rounded spots embedded in the microstructural features.

**Figure 4:**
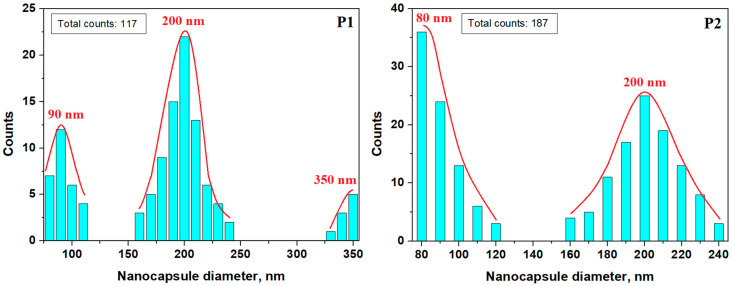
Nanocapsule diameter distribution for P1 and P2 samples.

The AFM topographical view of the ultrastructure in Figure 3b’ reveals well-individualized spherical nanocapsules with diameters ranging from 80 nm to 240 nm. The topographical aspect is better observed in the 3D profile that correlates with the mean surface roughness of Ra = 39.3 nm and Rq = 49.3 nm. The presence of the intermediary layer between the nanocapsules within the P2 sample causes a lower roughness than the one observed for P1. However, it is expected that an intensive physical treatment will break the thin film of the dispersion environment and will set the nanocapsules free. Both topographical AFM images in Figure 3a’,b’ were subjected to a particle diameter distribution and the obtained histograms are presented in Figure 4.

The mean diameter of the P1 sample is about 200 nm, but we observed some representative subgroups of nanocapsules of 90 nm and a small number of larger one of about 350 nm. The P2 sample has two dominant particle sizes, with small nanocapsules of 80 nm accompanied by a significant number of nanocapsules of about 200 nm. The distribution differences between the P1 and P2 samples are induced by the increased centrifugation speed of sample P2.

**Figure 5:**
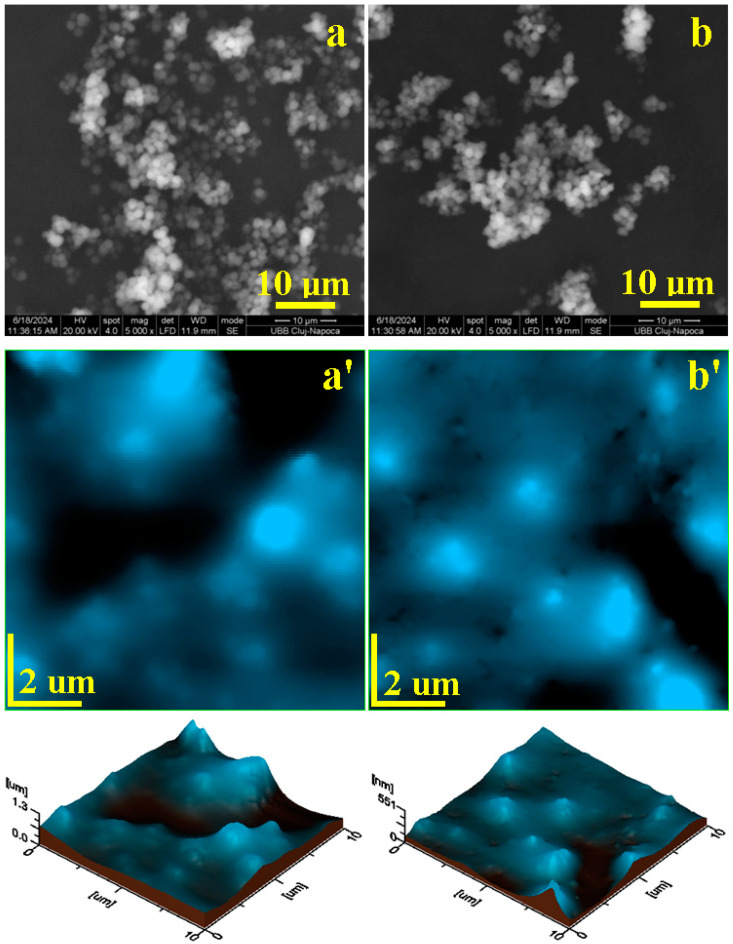
Microstructural investigation of nanocapsules after centrifugation: SEM images of samples P3 (**a**) and P4 (**b**). Microstructure accompanied by ultrastructure as revealed by AFM: samples P3 (**a’**) and P4 (**b’**). Topographic 3D profile is presented below each AFM image.

Increasing the stirring speed to 800 rpm ensures a better concentration of the core materials and a better shell formation. Thus, the SEM images in Figure 5a,b reveal bigger nanocapsules of about 800 nm with a spherical morphology, which are completely free without any bonding involved between them. The differences induced by the filtration process include the following: vacuum filtration induces a slight coalescence tendency, as seen in Figure 4, while spraying on the filter paper ensures a better individualization of the synthesized microcapsules.

The topographic image obtained for the sample P3, shown in Figure 5a’, reveals the microsphere deposits that resulted from the vacuum filtration. The agglomeration tendency is better observed in the median right side and top of the image, while the bottom area shows well-individualized microcapsules of about 800 nm. The distribution of larger microcapsules on the flat surface leads to an increased roughness value of Ra = 174 nm and Rq = 229 nm.

However, the coalescence tendency is significantly diminished by the spraying process, which forces the microcapsules to settle into distinct places, as observed in the topographic image Figure 5b’. The microcapsule diameter is constantly situated around 800 nm. The uniform distribution of the microcapsules affects the surface roughness as follows: Ra = 42.7 nm and Rq = 60.8 nm.

### 3.3. Controlled Release of the Active Compound

**Figure 6:**
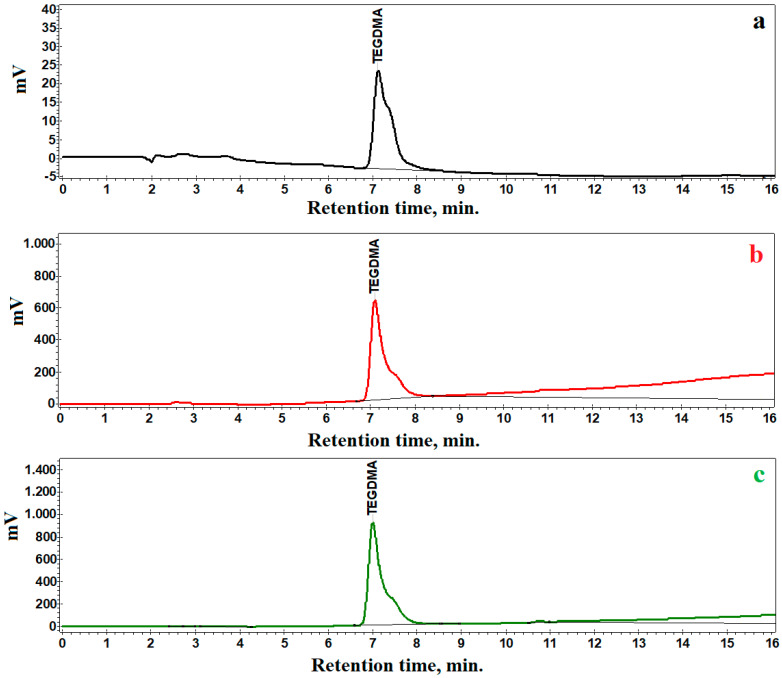
HPLC chromatograms obtained for (**a**) TEGDMA standard, (**b**) sample P2 after 7 days and (**c**) sample P3 after 14 days.

The micro and nanostructural investigation shows that the P2 and P3 samples have the best uniformity and stability, therefore they were subjected to the HPLC investigation. TEGDMA is the active compound within the liquid core of the microcapsules. Thus, it is assumed that HPLC chromatography is able to measure the released TEGDMA compared to the standard etalon. Figure 6a presents the chromatogram obtained for the TEGDMA etalon.

The progressive release of the TEGDMA is evidenced by the HPLC chromatograms for P2 after 7 days, shown in Figure 6b, revealing an increased peak compared to the standard. The TEGDMA peak progressively increases after 14 days, as seen in Figure 6c. This indicates a greater cumulative release of the active ingredient. Sample P3 has a similar behavior. The determined values of the released TEGDMA amounts are presented in Table 2.

It follows that, overall, both samples P2 and P3 have similar release behaviors. Sample P3 presents with slightly increased TEGDMA release after 7 days compared to P2, but it finally features an increased release after 14 days.

### 3.4. Low-Field NMR Investigations

**Figure 7:**
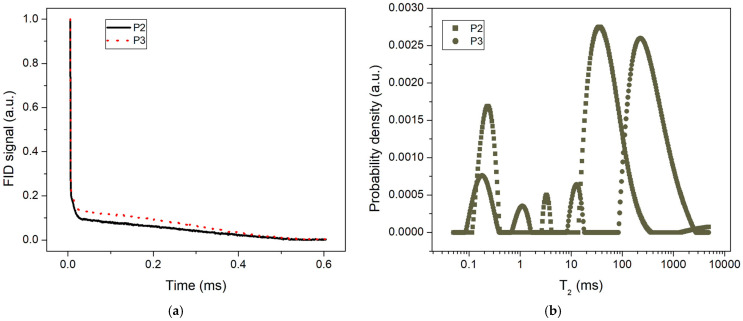
(**a**) The normalized FID signals recorded immediately after a 90-degree radiofrequency pulse. The steep decay corresponds to the solid-like component, and the slow decay to the liquid component. (**b**) The relaxation time distributions in the case of two samples (P2, P3) extracted via the CPMG technique. The largest peak corresponds to the liquid component inside the nanocapsules, and the smaller peaks may be associated with restricted molecular mobility as in solid-like components.

Figure 7a shows the relaxation time distributions extracted from the CPMG echo trains recorded in the case of the P2 and P3 samples. The observed distributions indicate the presence of a liquid component within the samples. Thus, peaks associated with mobile liquid molecules were detected at 34 ms (P2 sample) and 230 ms (P3 sample). The longer relaxation time for the liquid component in P3 compared to P2 suggests the presence of larger capsules within the P3 sample. The smaller peaks, with shorter relaxation times (<20 ms), correspond to less mobile molecules, possibly confined within nanostructured pores, or to nanocapsules that are smaller than those visible in the SEM analysis.

To estimate the liquid-to-solid ratio of the prepared sample, free induction decays (FIDs) following a 90-degree radiofrequency pulse were recorded, as shown in Figure 7b. These FIDs indicate the presence of two distinct components: a solid-like component characterized by a rapid decay of the FID signal and a liquid-like component distinguished by a slower decay. The solid-like component is likely represented by the capsule wall and other solid-like structures within the sample.

The liquid component encompasses both the healing agent confined within the nanocapsules and the interstitial space between the capsules. By analyzing these components at an evolution time of 5.4 µs, we can estimate the ratio between the liquid-like and solid-like components within the samples. This analysis reveals a liquid-to-solid ratio of 0.11 for the P2 sample and 0.16 for the P3 sample. This result, even if not very accurate, because it does not quantify the components with very short relaxation times, still indicates that sample P3 is more efficient in incorporating the healing agent.

## 4. Discussion

The amount of core content present in the microcapsule is determined by the parameters utilized to create self-healing capsules, such as the temperature, surfactant type, core-to-shell ratio, and stirring speed. The structural homogeneity and the effectiveness of the self-healing process are actually determined by the thickness of the capsule shell. Pittala and Ben investigated how the formation of microcapsules was affected by two distinct surfactants (polyvinyl alcohol and Tween 80), both separately and in combination. They investigated how the type and concentration of surfactant affected the microcapsules’ shell thickness. They came to the conclusion that, at a stirring speed of 500 rpm, a shell concentration of 0.5 w% and a surfactant (PVA) concentration of 3 w% could produce microcapsules with a size of 11.2–19.04 μm, a core-to-shell ratio of 4:1, and a temperature of 40 °C [38].

Changing the core-to-shell weight ratio has a direct impact on microcapsule size. The volume of the core material and the overall size of the microcapsules are greatly increased when there is a larger quantity of core material present since this results in a wider core droplet size within the emulsion. In UF in situ polymerization, an optimal ratio of 6.2/1 is frequently employed. This ratio improves the mechanical characteristics of the base sample by making it easier to produce spherical, well-formed microcapsules. On the other hand, too much core material results in poorer dispersion and less oligomer available to create a strong shell surrounding the core. As a result, there are far fewer microcapsules produced, and the final microcapsules have thinner, more fragile shell walls [14].

As a healing liquid, we chose TEGDMA because it is a fluid monomer that can easily penetrate into micro-cracks and quickly polymerizes, under the action of the initiating system for polymerization, which has been used for a long time in dentistry as a dilution monomer in the polymer matrix of composites [39]. Its presence inside the microcapsules was demonstrated both by the recorded FIDs and NMR relaxation time distributions.

The major challenge of composites with microcapsules is that the microcapsules in the composite can break when they encounter a micro-crack and can release the healing liquid inside them. A very important aspect for fracturing the microcapsules lies in the thickness of the coating that surrounds the healing liquid. If it is too thick, there is a possibility that the capsules will not break in contact with the crack, and if it is too thin, the capsules will break faster, being very fragile [39,40].

It has been reported that sizes of 24–70 μm would be indicated for microcapsules to ensure a crack of the shell and efficient delivery of the healing agent. Increasing the size of the capsule can negatively influence the composite, through the cross-linking of the resin matrix, followed by a decrease in mechanical properties. Also, increasing the size of the capsule can reduce the homogeneity within the capsules.

The microstructural analyses effectuated with SEM and AFM reveal that lower stirring speeds (400–600 rpm) during microcapsule formation result in a heterogeneous distribution containing some misshapen debris being observed, which requires centrifugation for removal. Thus, the P1 and P2 samples have rounded shapes and their size varies from 80 to 800 nm. This is a nano to submicron dispersion, which is very useful for certain composites. Samples P3 and P4 prove to be more uniform with a spherical shape and size of about 800 nm. Their size proves to be optimal according to the literature [39,40,41].

It is important to achieve an interconnection at the interface between the microcapsules and the crack, as at the moment when the crack propagates in the matrix and meets the microcapsules, it breaks easily. If, on the other hand, the interface is smooth, the crack in the resin matrix can bypass the microcapsules without breaking their shell, thereby missing the crack healing aspect [41]. It is difficult to establish shell thickness; therefore, the release of the TEGDMA active compound was assessed by HPLC chromatography. The results showed that both P3 and P4 have similar behaviors, being able to release the active compound gradually. They were measured to have a release of about 0.400 mg/g after 7 days, which increases to about 0.600 mg/g after 14 days.

There are several processing parameters that significantly influence the quality of microcapsules, such as the shell thickness and final size, which are highly sensitive to these factors. These parameters also greatly impact healing efficiency.

The agitation rate significantly influences the size of microcapsules. The diameter of the microcapsules is crucial for the healing process, as it determines the amount of healing agent that can be delivered when cracks and ruptures occur. Higher agitation rates tend to produce smaller core droplets, leading to smaller microcapsules. Brown et al. demonstrated a log–log linear correlation between the average diameter and agitation rate [42]. Similarly, Yin et al. found that the final size of microcapsules is inversely proportional to the stirring speed used during the preparation of the epoxy emulsion [43].

Kosarli et al. [44] investigated the relationship between capsule size and healing efficiency. Their results confirmed that capsule size has an inverse effect on the reduction in mechanical properties but a direct relationship with healing efficiency. In other words, using relatively large capsules can enhance healing efficiency, but the capsule size must be sufficiently large to contain enough healing agent to effectively maintain the damaged structure.

## 5. Conclusions

The stirring process influences microsphere uniformity: at 500 rpm, this results in spherical shapes, with sizes ranging from 80 to 800 nm, and the misconfigured debris requires centrifugation to be removed. Centrifugation at 600 rpm proves to be more effective than at 400 rpm, assuring a better removal of the misconfigured polymer debris. The optimal formation of uniform microcapsules is achieved at a stirring speed of 800 rpm without centrifugation. The 800 nm nanocapsules were extracted from the supernatant by two cost-effective methods, such as vacuum filtration and spraying on the solid substrate. These capsules present with a better control of liquid release of about 0.400 mg/mL after 7 days, which increases to about 0.600 mg/mL after 14 days. The free induction decays (FIDs) were recorded to estimate the liquid-solid ratio in the samples. The liquid-to-solid ratio for sample P2 was found to be 0.11 and 0.16 for sample P3. Therefore, sample P3 is more effective in incorporating the healing agent.

## Figures and Tables

**Table 1:** Sample synthesis parameters.

Samples	Stirring			Centrifuging			Filtration	Drying	
	Speed, rpm	Time, min.	Temp., °C	Speed, rpm	Time, min.	Temp., °C		Time, h	Temp., °C
P1	500	4	40–55	400			gravitational	24	25
P2	500	4	40–55	600			gravitational	24	25
P3	800	4	40–55	none			vacuum	24	25
P4	800	4	40–55	none			sprayed on filter paper	24	25

**Table 2:** HPLC results.

Samples	Released TEGDMA Amount, mg/1 mg of Sample	
	7 Days	14 Days
P2	0.400	0.570
P3	0.414	0.610

## Data Availability

Data are included in this article.
